# Concerted action of gammaherpesvirus infection and moderate hyperoxia on lung structural development including effects on critical lung fibroblast functions in neonatal mice

**DOI:** 10.1186/s40348-026-00256-x

**Published:** 2026-08-12

**Authors:** Anne Hilgendorff, Anna M. Dmitrieva, Beatrix Steer, Misako Nakayama, Erika Gonzalez, Motaharehsadat Heydarian, Heiko Adler

**Affiliations:** 1 Institute for Lung Health and Immunity and Comprehensive Pneumology Center With the CPC-M bioArchive, Helmholtz Zentrum München, Munich, Germany; 2 Institute of Asthma and Allergy Prevention, Helmholtz Zentrum München, German Research Center for Environmental Health, Neuherberg, Germany; 3 Walther Straub Institute of Pharmacology and Toxicology, Ludwig-Maximilians-University Munich, Munich, Germany; 4 Member of the German Center of Lung Research (DZL), Munich, Germany; 5 Dr. Von Hauner Children’s Hospital, Hospital of the Ludwig-Maximilians-University Munich, Munich, Germany; 6 Department of Paediatrics, Section of Neonatology, Pediatric Intensive Care, Pediatric Cardiology, Pediatric Pneumology and Allergology and Research Centre Neurosensory Science, School of Medicine and Health Sciences, Carl Von Ossietzky University Oldenburg, Oldenburg, Germany

**Keywords:** BPD, Viral infection, MHV-68, Fibroblasts

## Abstract

**Background:**

The development of chronic lung disease, also known as Bronchopulmonary Dysplasia (BPD), is the most prevalent complication after premature birth. Viral infections are discussed to drive or aggravate lung disease, but only little is known about their role in BPD pathophysiology. We therefore used a preclinical model as well as primary neonatal mouse lung fibroblasts to study the impact of viral infection on disease development in the presence or absence of hyperoxia induced lung injury.

**Methods:**

Neonatal C57BL/6 mice (PND 5–7) were infected with murine gammaherpesvirus-68 (MHV-68) by intranasal inoculation with or without pre-exposure to moderate hyperoxia (40%, 24 h). Next to the assessment of infection and replication (lytic and latent phase), lung histology and growth factor signaling were investigated together with indicators of proliferation and apoptosis early and late after infection. In vitro, these experiments were mirrored by exposing hyperoxia challenged and unchallenged primary neonatal mouse lung fibroblasts to MHV-68 (24 h). Analyses included cell function and developmentally relevant signaling.

**Results:**

MHV-68 infection in neonatal mice resulted in acute lung inflammation early after infection, followed by histopathologic abnormalities at 5 months of age and altered growth factor signaling in hyperoxia and virus exposed mice. In primary neonatal mouse lung fibroblasts, MHV-68 infection impaired critical developmental functions including migration and proliferation together with a decrease in *Pdgfr*-α expression that was reproduced in vivo in the lung periphery. Interestingly, in vitro virus-induced effects were only partially augmented by pre-exposure to clinically relevant levels of hyperoxia.

**Conclusions:**

The present study highlights the potential of MHV-68 to perturb fibroblast functions with a critical role in structural lung development. These effects are virus-dependent with longer-term effects being partially augmented by oxygen exposure. Our pilot study is the first that combines a relatively mild virus infection with exposure to only moderate levels of oxygen and underscores the importance to identify and better understand early, pathophysiologically relevant insults that impact on the dynamics of lung development and the need to verify these drivers in clinical studies.

**Supplementary Information:**

The online version contains supplementary material available at 10.1186/s40348-026-00256-x.

## Background

In the growing population of preterm neonates, chronic lung disease (Bronchopulmonary Dysplasia, BPD) remains the most prevalent complication, defining long-term pulmonary health and risk for comorbidity development [1, 2]. At the same time, BPD is a prime example when studying the impact of early environmental insults on the dynamics of organ growth and development with long-term consequences persisting into adulthood.

Although viral infections with respiratory syncytial virus (RSV) or human metapneumovirus and human rhinovirus (HRV) are known to affect infants with BPD, especially during the first years of life [3, 4], the impact of early postnatal virus exposure on the onset of BPD in concert with other, clinically prevalent mechanisms of lung injury remains largely unexplored.

In children and adults with chronic lung diseases such as asthma and chronic obstructive pulmonary disease, viral infections of the respiratory system have been identified as a risk for disease progression and lung function decline [5]. Likewise, herpesviruses are known to impact pulmonary development next to their well described adverse effects on the central nervous system, resulting in lung fibrosis and emphysema, with infants born premature or with low birth weight being specifically at risk for complications [6–9].

Pathophysiologically, viruses induce direct tissue injury resulting in reduced tissue repair capacities and DNA damage together with a sustained immune response, believed to be mostly mediated by infected epithelial cells and cells of the innate and adaptive immune system. In line with this, studies demonstrated the T-cell dependent induction of pulmonary fibrosis in mice following herpesvirus infection [10]. While alveolar epithelial cells are known to contribute to the virus-induced pulmonary immune response, in part depending on their capacity to present antigen recognition molecules [11], actions on lung fibroblasts are primarily described as a subsequent event resulting from proinflammatory cytokine expression derived from epithelial or other immune competent cells [12]. Cytokines mediating virus-dependent effects include TGF-beta, TNF-alpha, IL-1, IL-4, IL-5, IL-6, IL-13, and IL-17. With very little studies in the field addressing the effects of virus exposure on the developing lung in early postnatal life, insight on potential direct effects of viral infection on the (myo)fibroblast driving alveolar septation is lacking.

When aiming for study results with translational potential, the possible interplay of different drivers in BPD development acting on the immature lung has to be considered [13]. In addition to challenges imposed by perinatal infections, premature neonates often require life-saving oxygen therapy. Although survival improves with oxygen supplementation, morbidity increases with higher concentrations and longer exposures due to the proinflammatory effects paired with the induction of oxidative stress, apoptosis and structural remodeling [14, 15]. The induction of lung injury even by short term postnatal hyperoxia has been suggested by clinical observations [16–18], and is supported by experimental studies [19, 20]. Pathogenic viruses exhibit varying adaptability to oxygen levels to perform efficient host infection [21, 22]. In pediatric and adult patients, numerous investigations have previously confirmed that elevated oxygen levels are linked to harmful consequences, encompassing disruptions in immune responses, irregular metabolic function, and changes in both hemodynamics and alveolar barrier function [23, 24]. Together, this led to the association of oxygen treatment with the deterioration of lung damage and the advancement toward the onset of severe Acute Respiratory Distress Syndrome (ARDS) in COVID-19 patients [25].

To address the impact of early postnatal viral infections on critical lung fibroblast functions during alveolarization, we used murine gammaherpesvirus-68 (MHV-68) infection in a preclinical neonatal mouse model of lung disease combined with in vitro studies in primary neonatal lung fibroblasts to assess immediate and long-term effects. In a subsequent step, we advanced these studies into a double-hit model addressing the potential induction of vulnerability in these cells by pre-exposure to clinically relevant, moderate levels of hyperoxia, as the most prevalent postnatal treatment in neonates. For comparison to hyperoxia, we used TGFβ stimulation in vitro as a common injury response and driver of pathology in lung diseases in neonates [20].

## Methods

### In vivo experiments

#### Hyperoxia exposure in neonatal mouse pups

Newborn mice of the C57BL/6 wild-type (WT) strain, aged between five to seven days and born at term gestation [with a body weight of 3.6 ± 0.3 g], were randomly assigned to two experimental groups (*n* = 6–8 mice/group): Newborn mice and their mothers were either exposed to oxygen (FiO_2_ = 0.4) using the A-Chamber (Oxygen controller ProOx110, Biospherix, Parish, NY) or kept at room air (RA; FiO_2_ = 0.21) for 24 h.

#### MHV-68 infection

Eight days after exposure to hyperoxia or room air, mice were anesthetized with 2.5 μl/g of an antagonizable anesthetic solution containing 1 mg/ml Medetomidine, 5 mg/ml Midazolam, and 0.05 mg/ml Fentanyl and randomized to receive MHV-68 (1 × 10^4^ PFU in 10 μl of PBS) or PBS only intranasally. Following infection, anesthesia was reversed using 8.5 μl/g of the corresponding antagonist solution (5 mg/ml Antipamezole, 0.1 mg/ml Flumazenil, and 0.4 mg/ml Naloxone). At 5–6 months of age, all treatment groups exhibited comparable body weights with no indication of failed growth (Supplementary Fig. 3).

#### Organ harvesting

**Fig. 1:**
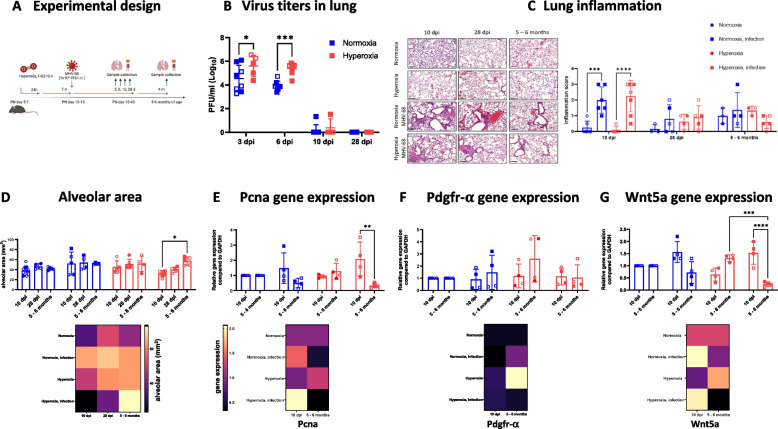
MHV-68 infection drives lung inflammation in neonatal mice and results in persisting histopathologic changes. **A** Scheme of the in vivo experimental design. 5–7 days-old mice pups were treated with O_2_ (FiO_2_ = 0.4) or room air (normoxia). Eight days after the first hit, the second hit was induced by infecting mice intranasally with MHV-68. Lungs, brains, spleens, and blood were harvested for analysis on 3, 6, 10, 28 days, or 4–6 months after the second hit (Picture was created with BioRender.com). **B** Virus titers in the lung. Virus titers were determined by Plaque assay in the right lung lobe of female (*n* = 3–4) and male (*n* = 2–4) mice. Statistical analysis: two-way ANOVA test with correction for multiple comparisons. *p < 0.05, ***p < 0.001. **C** Left panel: H&E staining of lung sections. Normoxia control mice, hyperoxia-exposed mice, MHV-68-infected mice, and both hyperoxia-exposed and MHV-68-infected mice on days 10, 28, and 5–6 months after infection. Scale bars: 100 μm. Right panel: Assessment of inflammation in H&E-stained lung tissue sections. A total lung inflammation score was determined for 3–6 mice in each group and graded on a scale of 0 (normal) to 3 (severe). Lung sections comprising all lung lobes were scored as a consensus by two scientists blinded to the groups. **D** Alveolar area, determined from mice after hyperoxia exposure and MHV-68 infection (*n* = 2–6 for female mice, and *n* = 1–4 for male mice). H&E-stained lung histology slide images were converted into Bioquant software images. Three to four non-overlapping 100 µm fields were sampled from each section, intentionally avoiding the large airways and blood vessels. All the alveoli were counted and measured with the help of software. Two-way ANOVA test with multiple comparisons corrections. *p-value < 0.05. **E** *Pcna* gene expression (*n* = 2 for both female and male mice). QPCR was used to quantitate the mRNA expression of *Pcna* in whole lung lysates from FiO_2_ = 0.4-treated and virus-infected mice and controls harvested 10 days, 28 days, and 5–6 months after treatment. *Gapdh* was used as a reference gene. Two-way ANOVA test with multiple comparisons corrections. *p-value < 0.05, **p-value < 0.01. **F** *Pdgfr-α* gene expression (*n* = 2 for both female and male mice). QPCR was used to quantitate the mRNA expression of *Pdgfr-α* in whole lung lysates from FiO_2_ = 0.4-treated and virus-infected mice and controls harvested 10 days, 28 days, and 5–6 months after treatment. *Gapdh* was used as a reference gene. Two-way ANOVA test with multiple comparisons corrections. **G** *Wnt5a* gene expression (*n* = 2 for both female and male mice). QPCR was used to quantitate the mRNA expression of *Wnt5a* in whole lung lysates from FiO_2_ = 0.4-treated and virus-infected mice and controls harvested on 10 days, 28 days, and 5–6 months after treatment. *Gapdh* was used as a reference gene. Two-way ANOVA test with multiple comparisons corrections. *p-value < 0.05, **p-value < 0.01. Data are always represented as mean ± SD. The lower panels in D) to G) display the corresponding data as heatmaps to illustrate overall patterns. dpi: days post infection; open symbols: females; closed symbols: males

All animals were euthanized at designated time intervals post infection (Fig. 1A), via injection of a lethal dose of pentobarbital and cervical dislocation. Lungs and spleens were dissected, placed in sterile Eppendorf tubes, and stored at −80˚C.

#### Lung fixation

Lungs were fixed intratracheally with 4% buffered PFA at a standardized pressure of 20 cm H_2_O as published previously [20].

#### Haematoxylin–Eosin staining

Formalin-fixed paraffin-embedded (FFPE) lung tissue sections from mice subjected to hyperoxia and controls, as well as those exposed to both hyperoxia and virus alongside controls, underwent an initial incubation at 60 °C for one hour, followed by tissue deparaffinization. The slides containing the tissue sections then underwent a sequential process involving two incubations in Xylene for 5 min each, followed by incubations in 100%, 90%, 80%, and 70% ethanol for 2 min each. This was succeeded by a 5-min incubation in 1xPBS. Subsequently, the slides were stained with Hematoxylin Mayer for 10 min. After rinsing with tap water, the slides were immersed in a 1% Eosin solution (Carl Roth) for 1 min and then washed in VE water. The slides were then dehydrated through successive immersions in 80% ethanol, 90% ethanol, 100% ethanol, and Xylene (5 min each). Finally, coverslips were mounted using a Xylol-based Fast Mounting Medium (Biosystems).

#### Lung histology

Following isotropic uniform, random (IUR) sectioning [26], quantitative assessments of the alveolar area (referring to the individual alveolar size) (≥ 30 fields of view; 400X magnification) were conducted on 2–3 independent random 4 μm H&E tissue sections per animal using CAST-Grid 2.1.5 (Olympus) [27]. A lung inflammation score was determined using a single section of all lung lobes for 3–6 mice of each group and graded on a scale of 0 (normal) to 3 (severe) (consensus scoring two scientists blinded to the groups), scoring the presence of inflammatory cells both around airways or vessels and in the alveolar space or interstitial tissue. Additional morphometric analyses performed in our study included tissue area (total area covered by tissue per field of view selecting peripheral lung tissue, excluding large vessels and airways), airspace area (referring to the individual airspace), and the microvessel number.

#### Immunofluorescence staining of paraffin-embedded tissue sections

For immunofluorescence, sections were treated with 0.1 M citric buffer, pH 6, and heat ≥ 96 °C for antigen retrieval followed by tissue permeabilization (Triton X-100 (0.5%)). Antibody incubation was performed at 4 °C overnight with anti-Pdgfr-α (Sc-338, Santa Cruz Biotechnology), followed by secondary antibody incubation with an Alexa Fluor 568 (Molecular Probes; Eugene, OR; 1 h), counterstaining with DAPI (Sigma Aldrich #D8417) and mounting using Dako (Agilent, Santa Clara). The number of total nuclei as well as single- or double-positive cells were quantified at 400 × magnification in the lung periphery excluding large airways and vessels, processed by FIJI [28].

#### Determination of lung viral titers

To determine lytic virus titers, lungs were harvested at the indicated time points after infection (Fig. 1B) and homogenized by using the FASTPREP − 24 instrument (MP Biomedicals, Heidelberg, Germany). After two times freezing and thawing the homogenates, plaque assays were performed with ten-fold dilutions of the supernatants on BHK-21 cells as described previously [29].

#### Determination of viral load in spleens

Viral load in splenocytes of infected mice was determined by quantitative real-time PCR using the ABI 7300 Real Time PCR System (Applied Biosystems, Foster City, CA) as described previously [30].

### In vitro studies

#### Isolation of mouse primary fibroblasts from neonatal lungs

Following injection with a lethal dose of pentobarbital, lungs were harvested from 5- to 7-day-old neonatal mice. The lungs were flushed with 1X PBS through the right ventricle until they appeared almost white, then removed. The lung lobes were dissected and placed in a 6-well plate containing ice-cold 1X PBS. The lung tissue was then cut into 1 mm pieces using a scalpel and placed in 500 µL of PBS. The dishes were left to dry at 37 °C for about 15 min. Once the tissue pieces adhered to the dish surface, 8 mL of DMEM-1 medium (Gibco, Darmstadt, Germany) supplemented with 10% fetal bovine serum (FBS) and 1% penicillin–streptomycin was slowly added. Two to three days later, the medium and any floating tissue pieces were removed, and 10 mL of fresh medium was added. Experiments were initiated once cell confluence reached 70–80%. Isolated cells were characterized with a fluorescence‐activated cell sorter (FACS LSRII) using multicolor staining of CD45 (BD Pharmingen), CD105 (BD Pharmingen), CD90 (BD Pharmingen), PDGF‐Rα (eBiosciences), Vimentin (Cell Signaling), and α‐SMA (R&D systems) as published previously [20]***.***

### In vitro hyperoxia exposure

At a confluence of > 90%, cells were seeded in 6-well plates and after reaching optimal confluence exposed to either hyperoxia (FiO_2_ = 0.4) or kept at room air (FiO_2_ = 0.21). To optimize oxygen diffusion in culture [31], we exposed monocultures on 100 mm dishes, each with a surface area of 56.7 cm^2^, containing 10 ml of culture medium as published previously [32]. Oxygen was supplemented using a Biospherix ® (Oxygen controller ProOx110, Parish, NY) standard set up with a routinely calibrated oxygen sensor in combination with the incubator subchamber system that provides O_2_/CO_2_ setpoint control.

### TGFβ treatment

Primary fibroblasts were treated for 24 h at 37° degrees with 5 ng/ml of TGFβ (R&D Systems).

### MHV-68 infection of primary lung fibroblasts

#### Virus stock preparation

MHV-68 was grown and titrated on BHK-21 cells as previously described [29].

#### Infection of cells

Following treatment, cells were washed twice with PBS and subsequently incubated with the virus solution for 1 h at 37 °C. The inoculum was then replaced with an equivalent amount of fresh medium. Infected cells were further incubated at 37 °C for the indicated times before either harvesting the cells or conducting functional analysis.

#### Determination of infection rate

Neonatal mouse lung primary fibroblasts were infected with a recombinant MHV-68 expressing GFP [29]. 24 h after infection, the medium was discarded, and flasks/dishes were washed with PBS. Subsequently, trypsinization was carried out using a pre-warmed 0.25% Trypsin–EDTA (Gibco) solution for 5 min at 37 °C. The cells were then centrifuged at 8000 g for 5 min at 24 °C, resuspended in PBS, and centrifuged again. The resulting pellet was dissolved in 100 μl of 2% PFA in PBS and transferred to FACS tubes. Acquisition of infected cells was performed using BDTM LSRFortessa with BD FACSDivaTM software version 8.0, and subsequent analysis for GFP expression was conducted using Flowing Software 2.5.1.

#### Determination of viral replication

Cells were first treated with TGFβ or exposed to hyperoxia for 24 h. Subsequently, cells were infected with an MOI of 0.1 for 1 h. After removing the inoculum, cells were incubated with fresh medium at 37 °C and 5% CO_2_ until the supernatants together with the cells were harvested at the indicated time points after infection. Virus titers were determined by Plaque assay.

#### Trypan blue cell counting

After a 24-h treatment and infection period at 37 °C with 5% CO_2_, cells were harvested using Trypsin–EDTA (Gibco), centrifuged and reconstituted in 1 ml of fresh medium. Viable cells were counted using 0.4% Trypan Blue Solution (Gibco).

#### Wound healing "scratch" assay

For the wound healing scratch assay, cells were plated in 6-well plates. Following treatment with TGFβ or exposure to hyperoxia, cells underwent infection, and an artificial scratch was manually created using a 200 μl tip. Subsequently, the cells were washed with PBS to eliminate any cell fragments or detached cells, covered with culture medium, and an immediate microscope image was captured. The cells were then incubated for 24 h (at 37 °C, 5% CO_2_), and another image was captured. Using the software "ImageJ", the scratch area was quantified at each time point and under each condition. Subsequently, the wound closure was determined through the following calculation: Wound closure, % = (initial scratch area, 0 h—scratch area at 24 h) × 100%, devided by initial scratch wound area, 0 h.

#### Cell migration/chemotaxis assay

The assay was conducted following the manufacturer’s guidelines using the EZCellTM Cell Migration/Chemotaxis Assay Kit, 8 μm (K906-100; BioVision). The fluorescence signal was recorded using a GloMax® Explorer Multimode Microplate Reader (Promega) at 530 nm emission light/590 nm excitation light. Alternatively, the assay was conducted utilizing the CytoSelect 96-well Cell Migration Assay Kit, 8 μm, Fluorometric Format (ab235673; Cell Biolabs) following the guidelines provided by the manufacturer. In that case, the fluorescence signal was detected using a GloMax® Explorer Multimode Microplate Reader (Promega) at 480 nm emission light/520 nm excitation light.

#### Caspase-Glo 3/7 assay

The experiment was carried out following the guidelines provided by the manufacturer (Promega). The luminescence signal was detected on the GloMax® Explorer Multimode Microplate Reader (Promega) using the built-in program.

#### RealTime-Glo Annexin V apoptosis and necrosis assay

The experiment was conducted following the manufacturer's protocol (Promega). The luminescence and fluorescence signals were detected on the GloMax® Explorer Multimode Microplate Reader (Promega) following the plate reader's built-in program at the time points 24 h, 48 h, and 72 h after infection.

#### MTT assay

MTT Solution (Sigma-Aldrich) was prepared according to the manufacturer's instructions. Cells were seeded in a 96-well plate at a density of 10,000 cells per well in a volume of 100 μl, followed by the administration of the treatment. After 24 h of incubation, 20 μl of the MTT solution was added to each well, and the plate was incubated at 37 °C for 4 h. Wells containing 0.1% DMSO and medium were used as negative controls, while wells containing only medium served as blank controls. After incubation, the medium was carefully aspirated, and the formazan crystals formed were dissolved in 100 μl of MTT solvent. Absorbance was then measured at 570 nm using a Tecan ELISA Reader (Life Sciences).

#### Protein isolation, SDS-PAGE, and immunoblotting

Cells cultured in 6-well plates were washed twice with sterile PBS and trypsinized using 0.25% Trypsin. The cell pellet was resuspended in 150 μL of RIPA lysis buffer with a Halt protease inhibitor cocktail (Thermo Fisher Scientific) incubated on ice for 30 min, and then centrifuged at 15,000 RPM for 15 min at 4 °C to separate the supernatant (total protein). The supernatants were stored at −80 °C. Protein concentrations were determined using the BCA assay (Thermo Fisher Scientific). The protein samples were prepared with NuPAGE® Reducing Agent, NuPAGE® LDS Sample Loading Buffer, and deionized water, then subjected to SDS-PAGE using Tris–Acetate, Tris–glycine, or Bis–Tris gels (Life Technologies) at 80–120 V. Proteins were transferred to Nitrocellulose membranes at 30 V for 90–100 min. Membranes were blocked with 5% milk in 1X TBS-T (0.1% Tween®20 in TBS) for 120 min and incubated overnight with primary antibodies at 4 °C. Membranes were incubated with HRP-conjugated secondary antibodies for 2 h at room temperature. Protein detection was performed using Femto SuperSignal® chemiluminescent substrates (Thermo Fisher) on a Chemidoc XRS + system (Bio-Rad). Primary and secondary antibodies used included B-Actin-HRP (ab49900; Abcam), PDGF‐Rα (C‐20, Santa Cruz Biotechnology #338) and Anti-rabbit IgG-HRP (sc-2357; Santa Cruz).

#### RNA-isolation from cultured cells and RT-PCR

mRNA was isolated from cells using the Nucleospin RNA Plus Kit (Macherey–Nagel, Dueren, Germany). Then, 1 µg RNA was reverse-transcribed using the High Capacity cDNA Reverse Transcription Kit (Applied Biosystems, Foster City, CA, USA) according to the instructions of the manufacturer. Subsequently, 2 μl of the resulting cDNA (or water as non-template control) were used as a template for PCR amplification with the Light Cycler 480II (Roche), using Light Cycler 480 SybrGreen I Master Mix (Roche) and universal cycling conditions (Roche). The following gene specific primers were used:

- GAPDH forward: 5’-AGGTCGGTGTGAACGGATTTG-3’
- GAPDH reverse: 5’-TGTAGACCATGTAGTTGAGGTCA-3’
- PDGFR-α forward: 5’-AGGTATGTATCCACACATGCGT-3’
- PDGFR-α reverse: 5’-AGTTCCTGTTGGTTTCATCTCG-3’
- PCNA forward: 5’-TTTGAGGCACGCCTGATCC-3’
- PCNA reverse: 5’-GGAGACGTGAGACGAGTCCAT-3’
- Wnt5a forward: 5’-GACTCCGCAGCCCTGCTTTG-3’
- Wnt5a reverse: 5’-CCAATGGGCTTCTTCATGGCGAG-3’

### Statistical analysis

Datasets were analysed using the GraphPad Prism software, vs9 (GraphPad Software, Inc., San Diego, CA, USA) by One- or Two-way ANOVA test with multiple comparisons correction. Results with a *p*-value < 0.05 were considered significant.

## Results

### MHV-68 infection induces lung inflammation and affects critical markers of organ growth

Newborn mice underwent intranasal inoculation with MHV-68 (1 × 10^4^ PFU, day 13–15 of life) with or without preceding exposure to moderate hyperoxia (O_2_; fiO_2_ = 0.4, day 5–7 of life, i.e. 8 days prior to infection). Mice were sacrificed at days 3, 6, 10, 28 (i.e. 16–43 days of life), and 5–6 months after virus infection (Fig. 1A). Mice from all study groups showed relevant pulmonary MHV-68 titers at three- and six-days post infection that were cleared at day 10 post infection in the majority of cases and no longer detectable 28 days post infection (Fig. 1B).

**Fig. 2:**
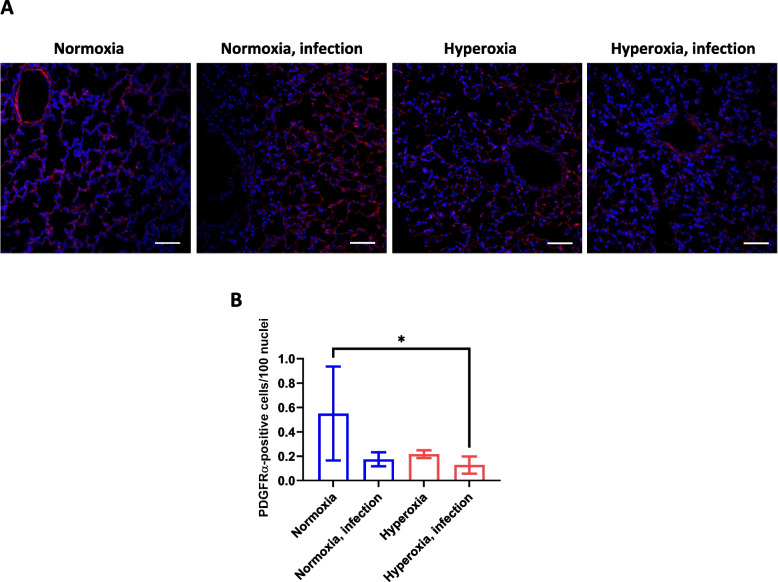
Hyperoxia plus infection decrease the number of PDGFR-α positive cells in neonatal mouse lungs. 5–7 days-old mice pups were treated with O_2_ (FiO_2_ = 0.4) or room air (normoxia). Eight days after the first hit, the second hit was induced by infecting mice intranasally with MHV-68. Lungs were harvested for analysis around 7 weeks after the second hit. **A** Representative images of immunofluorescence (IF) staining for PDGFR-α in lung tissue Sects. (4 μM, 400X). Nuclei are in blue (DAPI) and PDGFR-α positive cells are in red. The scale bar indicates 50 µm. **B** Quantitative analysis (quantification of IF in 10 fields of view (FOV) per section in 3 sections per animal, normalization of positive cells to 100 nuclei). Data are represented as mean ± SD. Statistical analysis: One-way ANOVA test with multiple comparisons corrections. *p < 0.05

Exposure to hyperoxia before infection resulted in elevated lung virus titers early after infection (days 3 and 6) (Fig. 1B). No difference was observed in the viral genomic load detected in the spleen at all time points analyzed (Suppl. Figure 1A). Lungs of mice infected with MHV-68 displayed histopathological signs of organ injury with peribronchial and interstitial thickening in the gas exchange area at day 10 after infection (Fig. 1C). The changes were less pronounced at later time points and were unaffected by the pre-exposure to hyperoxia (Fig. 1C). Whereas the lungs did not show significant changes in airspace area and tissue area at early time points (Suppl. Figure 1B and C), long term assessments, i.e. 5–6 months post-infection, revealed a significant increase in the alveolar area in mice exposed to hyperoxia and MHV-68 (Fig. 1D), pointing towards differences in the dynamics of structural development. These changes were accompanied by a decrease in gene expression indicating proliferation (*Pcna* expression) and epithelial cell development (*Wnt-5a* expression) (Fig. 1E and G). In line with this, immunofluorescence staining showed a significantly decreased number of PDGFR-α-expressing cells in the lung periphery in mice exposed to both hyperoxia and MHV-68 around 7 weeks after virus exposure (Fig. 2). Overall lung Pdgfr-α gene expression remained unchanged (Fig. 1F), indicating a functionally relevant down regulation in the lung periphery, ameliorated when assessing total lung tissue expression including Pdgfr-α of e.g. airway mesenchymal cells. Taken together, as summarized by the heatmaps (Fig. 1D-G, lower panels), virus exposure in the presence of hyperoxia resulted in distinct differences in developmental patterns at later time points indicated by histopathologic characteristics and lung growth factor expression.

Microvessel number in the gas exchange area was not significantly affected in the different study groups (Suppl. Figure 1D).

### MHV-68 infection impairs developmentally relevant functions in primary neonatal mouse lung fibroblasts

**Fig. 3:**
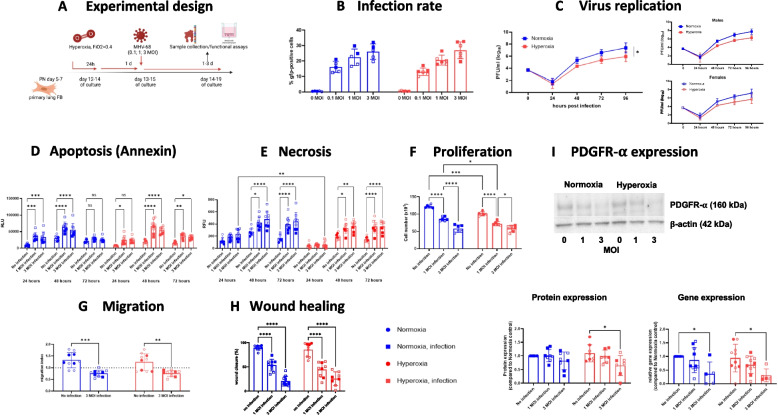
MHV-68 infection impairs critical developmental functions in primary neonatal mouse lung fibroblasts. **A** Scheme of the in vitro experimental design. Primary neonatal mouse lung fibroblasts were isolated from 5–7 days-old pups and after 12 to 14 days of culture, treated with O_2_ (FiO_2_ = 0.4) or room air (normoxia) for 24 h. One day later, the second hit was induced by infecting the cells with MHV-68. Analyses were performed one, two or three days after the second hit (Picture was created with BioRender.com). **B** MHV-68 infection rates in mouse primary lung fibroblasts with or without prior exposure to hyperoxia (FiO_2_ = 0.4) for 24 h. Primary neonatal mouse lung fibroblasts were isolated from the lungs of *n* = 3 female and *n* = 3 male newborn mice. Percentages of gfp-positive (= infected) cells are shown (mean ± SD). **C** Virus replication in mouse primary lung fibroblasts with or without prior exposure to hyperoxia (FiO_2_ = 0.4) for 24 h (left panel: both sexes combined; right panels: males and females separated). Primary neonatal mouse fibroblasts were isolated from the lungs of *n* = 5–6 female and *n* = 5–6 male newborn mice. Cells were infected with an MOI of 0.1, and virus titers were determined by Plaque assay at the indicated time points after infection (24 to 96 h). Titers at 0 h represent input inocula. A two-way ANOVA test with multiple comparisons correction was used for statistical analysis. *p-value < 0.05. **D** Annexin V binding after MHV-68 infection with or without prior exposure to hyperoxia. Primary neonatal mouse lung fibroblasts were isolated from the lungs of *n* = 6 female and *n* = 3 male newborn mice. Normoxia served as a control for hyperoxia exposure. RLU: relative luminescence units. Two-way ANOVA test with multiple comparisons correction. *p-value < 0.05, **p-value < 0.01, ***p-value < 0.001, ****p-value < 0.0001. **E** Necrosis after hyperoxia exposure (FiO_2_ = 0.4) for 24 h and MHV-68 infection for 24 h. Mouse primary fibroblasts were isolated from the lungs of *n* = 6 female and *n* = 3 male newborn mice. Normoxia served as a control for hyperoxia. RFU: relative fluorescence units. Two-way ANOVA test with multiple comparisons correction. *p-value < 0.05, **p-value < 0.01, ***p-value < 0.001, ****p-value < 0.0001. **F** Proliferation after 24 h of MHV-68 infection with or without prior exposure to hyperoxia of primary neonatal mouse lung fibroblasts. Cell numbers were determined by trypan blue staining and manual counting (*n* = 3 female and *n* = 3 male mice). A two-way ANOVA test with multiple comparisons correction was used for statistical analysis. *p-value < 0.05, ***p-value < 0.001, ****p-value < 0.0001. **G** Migration after 24 h of MHV-68 infection with or without prior exposure to hyperoxia. Primary neonatal mouse lung fibroblasts were isolated from the lungs of *n* = 6 female and *n* = 4 male newborn mice. Two-way ANOVA test with multiple comparisons correction. *p-value < 0.05, **p-value < 0.01, ***p-value < 0.001, ****p-value < 0.0001. **H** Wound healing (“scratch” assay) after 24 h of MHV-68 infection with or without prior exposure to hyperoxia. Mouse primary fibroblasts were isolated from the lungs of female (*n* = 3–6) and male (*n* = 3–4) newborn mice. Statistical analysis: Two-way ANOVA test with correction for multiple comparisons. *p < 0.05, **p < 0.01, ***p < 0.001, ****p < 0.0001. **I** PDGFR-α protein (lower left panel) and *Pdgfr-α* gene (lower right panel) expression after 24 h of MHV-68 infection with or without prior exposure to hyperoxia. Mouse primary fibroblasts were isolated from the lungs of newborn female (*n* = 4) and male (*n* = 3–4) mice. Protein expression was determined using protein lysates. A representative immunoblot is shown in the upper panel, and quantifications in the left lower panel. QPCR was used to quantify the mRNA expression of *Pdgfr-α* (*n* = 3–6 female and *n* = 2–4 male mice per group). Two-way ANOVA test with multiple comparisons correction was used for statistical analysis. *p-value < 0.05. Data are always represented as mean ± SD. dpi: days post infection; open symbols: females; closed symbols: males

Infection of primary neonatal mouse lung fibroblasts (day of life 5–7, female *n* = 3, male *n* = 3) with recombinant MHV-68 expressing GFP (0,1 MOI, 1 MOI, 3 MOI; control 0 MOI) (Fig. 3A) resulted in a significant, dose-dependent increase of GFP-positive cells with a maximum infection rate of 30% 24 h after infection (Fig. 3B). While this effect was unaltered by exposure to moderate hyperoxia, virus replication was reduced at later time points in hyperoxia-exposed cells (Fig. 3C, left panel). Gender-related differences were not observed (Fig. 3C, right panels).

MHV-68 infection of neonatal mouse lung primary fibroblasts resulted in increased apoptosis and necrosis 48 h (apoptosis, necrosis) and 72 h (necrosis) post-infection (Fig. 3D, E) without gender-related differences. In cells exposed to both hyperoxia and infection, apoptosis persisted 72 h after infection, when compared to cells undergoing virus infection only (Fig. 3D). The changes were found to be independent of Caspase 3/7 signaling (Suppl. Figure 1E), and indicators of cell metabolism remained unaffected by the exposures (Suppl. Figure 1 F).

MHV-68 infected primary neonatal lung fibroblasts showed a significant decrease in their capacity to proliferate and to migrate (Fig. 3F, G), resulting in their insufficient performance in a scratch wound-healing assay (Fig. 3H). These changes were associated with a change in cellular shape (Suppl. Figure 1G) and the decreased transcription of *Pdgfr-α,* known to drive these critical myofibroblast functions. Pre-exposure to hyperoxia augmented the changes observed (Fig. 3I). In contrast, pre-treatment of the primary neonatal lung fibroblasts with TGFβ, a prevalent cytokine expressed in the injured neonatal lung [20], did not potentiate the MHV-68 induced changes in viral infection rate and replication, apoptosis (except a slight increase after 24 h of infection with an MOI of 1), necrosis and wound healing (Suppl. Figure 2A-E).

## Discussion

Triggers of onset and progression in chronic lung disease during development remain of significant clinical and scientific interest as different treatment strategies did not alter overall disease incidence until today. Proven by clinical insight and experimental knowledge, the induction and progression of disease is multifactorial, making it necessary to engage multiple hit models in which inevitable environmental hits and inflicted treatment regimens act in concert to create clinically meaningful insight.

We therefore designed a preclinical double-hit mouse model to delineate the impact of the exposure to (clinically often undetected) virus infection in the presence or absence of moderate hyperoxia, the most prevalent postnatal treatment in neonates, on critical hallmarks of lung structural development.

Interestingly, we identified the early postnatal exposure to MHV-68 as a driver of lung injury during structural development, resulting in long-term effects when acting in concert with the exposure to moderate hyperoxia. As demonstrated by our in vitro experiments, the lung fibroblast functions as a direct target of MHV-68 that is commonly considered to act on immune-competent cells including B cells, macrophages, dendritic and epithelial cells. The infection of the neonatal primary lung fibroblasts resulted in the impairment of critical cellular functions relevant for alveolar septation. These in vitro changes were accompanied by the change in cell shape together with the significant reduction in Pdgfr-α expression, the driver of the myofibroblast’s migratory and proliferative capacity during lung development and repair [33]. Changes in Pdgfr-α expression were accompanied by impaired Wnt-5a expression in vivo and in vitro, which has been described in adult and neonatal lung injury including discussions about therapeutic options [34–36]. Research involving animals has indicated the importance of PDGF signaling in alveolarization, and it has been observed that the expression of PDGFR-α is diminished in mesenchymal stromal cells (MSCs) of neonatal lungs from infants who later develop bronchopulmonary dysplasia [37]. In line with this, exposure to severe hyperoxia (FiO_2_ > 0.7) resulted in a reduction of fibroblasts expressing PDGFR-α together with impaired canonical WNT/β-catenin signaling [34, 38]. Presenting previously unidentified immediate and sustained effects of early postnatal virus infection, we demonstrated impaired signaling through these pathways as a potential explanation for the observed impairment in alveolar septation. These effects occurred, however, independent of classical caspase-driven apoptosis or changes in indicators of cell metabolism.

As expected, lungs of mice infected with MHV-68 displayed histopathologic signs of organ injury during the acute phase of infection. This is consistent with previous observations where MHV-68 infection of the lung caused mild interstitial pneumonia [39], in contrast to e.g. influenza A virus infections, where mice develop significant lung disease [40]. In our model, initial inflammatory changes early after virus infection in vivo were followed by longer-term differences in lung developmental dynamics when exposure to virus infection and hyperoxia acted in concert, including changes in lung structure and growth factor signaling. This can be interpreted both as a direct effect on the cell as well as a cytokine mediated effect, triggered by both hyperoxia [20] or MHV-68 [41–43]. The structural changes are likely provoked by early developmental defects, reflected by the significantly decreased number of PDGFR-α-expressing cells in mice exposed to both hyperoxia and MHV-68 at around two months of life, consistent with the in vitro data. The lack of changes in total lung Pdgfr-α expression likely relates to gene expression in large airways but can as well be due to the time window studied. Clearly, future studies are needed to gain deeper insight into the cellular phenotypes affected by the virus in the developing lung as well as their subsequent interactions with neighboring cells populating the alveolar niche including both direct and indirect effects of the virus as well as the testing of different virus families.

We used MHV-68, a gammaherpesvirus that readily infects laboratory mice and reflects a natural pathogen-host scenario. Although the natural hosts of MHV-68 are wood mice, application of the virus to laboratory mice (Mus musculus) is a broadly accepted model of virus infection, presenting with a similar course of infection and subsequent disease and thus allowing its systematic examination in vivo [44]. Following intranasal inoculation, acute productive virus replication in the lung has been observed in macrophages and alveolar epithelial cells, resulting in peak lung titers at 4–7 days post infection before becoming largely undetectable by 9–12 days post infection. The primary pathology is mild interstitial pneumonia [39]. Following the acute phase, MHV-68 establishes a chronic lifelong infection, with B cells, macrophages, dendritic cells and alveolar epithelial cells harboring latent MHV-68 [44, 45]. The effect on lung fibroblasts, however, has not been studied in detail before.

When studying virus infection in the presence of a second environmental hit, we observed that exposure of primary mouse fibroblasts to hyperoxia prior to infection decreased viral replication in vitro. In contrast, exposure of neonatal mice to hyperoxia prior to infection resulted in elevated lung virus titers early after infection while it did not prolong the time to control the virus in vivo. Whereas our in vitro data relate to direct effects of both exposures on the cell in the absence of surrounding tissue and especially in the absence of (stimulated) immune cells, the contribution of these cell types in vivo is relevant and needs to be addressed in future studies. The effect of hyperoxia on cellular proliferation depends on cell type, cell cycle and compensatory effects including cellular crosstalk. Hyperoxia can directly limit the available “substrate” for virus replication, providing an explanation for the reduced virus titers in vitro, or act through an altered immune response since early life exposure to hyperoxia affects both innate and adaptive immune mechanisms [40], including an altered NK cell response to IAV infection [46] as well as impaired circadian AT2 cell functions [47]. Although we demonstrated augmented effects on virus replication as well as on impaired PDGFR-α expression in vivo and in vitro after preexposure to hyperoxia, detailed studies need to address underlying effects on the lung’s capacity for regeneration and repair. Interestingly, it has been described that low oxygen concentrations (hypoxia) accelerate lytic virus production of both MHV-68 and Epstein-Barr virus (EBV) [48, 49]. In case of MHV-68, this effect was linked to the induction of hypoxia-inducible factor 1 alpha (HIF1α) which promoted virus replication by impacting viral gene expression [48]. Future studies need to employ longer-term experiments to address conditions of (lung) virus reactivation during development [8]. As a limitation of our in vitro model that has been well established [20], primary cell cultures always bear the risk of phenotypic, functional and transcriptional changes in the cultured cell that are not fully accounted for.

Previous studies that investigated virus infection in the context of hyperoxia used Influenza A virus (IAV), respiratory syncytial virus (RSV) or Pneumonia virus of mice (PVM). While IAV replicates well in mice and models human disease, RSV, however, does not efficiently replicate in mice which limits the interpretation of these experiments [40]. In contrast to RSV, PVM is a natural pathogen in mice, thus allowing to study the infection in a natural pathogen-host context [50]. Nonetheless, the short-term exposure to moderate oxygen concentrations is less explored as other studies frequently used significant oxygen concentrations and or extended exposure times [51, 52]. Interestingly, our results obtained through mild virus infection and moderate levels of oxygen reflect findings from studies using PVM together with high oxygen concentrations[53], suggesting that priming of the lung for fibrotic repair by moderate hyperoxia holds underestimated clinical significance for subsequent virus infection [52].

## Conclusions

In summary, our study highlights the potential of MHV-68 to perturb critical fibroblast functions relevant in development and regeneration [19, 20, 54, 55], thereby adding to known detrimental virus effects mainly attributed to the epithelial cell [56]. The observed effects are virus-dependent and in part augmented by clinically relevant levels of oxygen. Future studies detailing these effects will likely support the clinical need to improve both monitoring and treatment of mostly undetected early postnatal virus infections in the vulnerable cohort of preterm infants, as these scenarios can account for adverse long-term pulmonary outcome [57]. Future studies should address the proliferative activity of specific cell types exposed to virus with and without preexposure to hyperoxia using clinically relevant settings regarding dose and timing. This could entail studies addressing the single cell level as well as spatial resolution to detail pathophysiologic insight and identify targeted treatment avenues.

## Supplementary Information

Supplementary Material 1.

Supplementary Material 2.

## Data Availability

The data generated and analyzed during the current study are included in this published article and its supplementary files and are available from the corresponding authors upon reasonable request.

## Abbreviations

BPD: Bronchopulmonary dysplasia
MHV-68: Murine gammaherpesvirus-68
PND: Postnatal day
