# Supplementary material for: Concerted action of gammaherpesvirus infection and moderate hyperoxia on lung structural development including effects on critical lung fibroblast functions in neonatal mice
Source: Mol Cell Pediatr. 2026 Aug 12;13:43. doi: 10.1186/s40348-026-00256-x (PMC13462024; doi:10.1186/s40348-026-00256-x)
Supplement: Supplementary file 1 [file 40348_2026_256_MOESM1_ESM.pdf]

Supplementary Information

Concerted action of gammaherpesvirus infection and moderate hyperoxia on lung structural development including effects on critical lung fibroblast functions in neonatal mice

Anne Hilgendorff [1,4,5]*, Anna M. Dmitrieva [1], Beatrix Steer [2], Misako Nakayama [1], Erika Gonzalez [1], Motaharehsadat Heydarian [1], Heiko Adler [2,3]*

[1]Institute for Lung Health and Immunity and Comprehensive Pneumology Center with the CPC-M bioArchive, Helmholtz Zentrum München, Member of the German Lung Research Center (DZL), Munich, Germany
[2]Institute of Asthma and Allergy Prevention, Helmholtz Zentrum München, German Research Center for Environmental Health, Neuherberg, Germany; Member of the German Center of Lung Research (DZL), Munich, Germany
[3]Walther Straub Institute of Pharmacology and Toxicology, Ludwig-Maximilians-University Munich, Munich, Germany; Member of the German Center of Lung Research (DZL), Munich, Germany
[4]Dr. von Hauner Children's Hospital, Hospital of the Ludwig-Maximilians-University Munich, Munich, Germany
[5]School of Medicine and Health Sciences, Department of Paediatrics, Section of Neonatology, Pediatric Intensive Care, Pediatric Cardiology, Pediatric Pneumology and Allergology and Research Centre Neurosensory Science, Carl von Ossietzky University Oldenburg, Germany

* Corresponding authors:
Anne Hilgendorff: anne.hilgendorff@med.uni-muenchen.de
Heiko Adler: heiko.adler@lrz.uni-muenchen.de

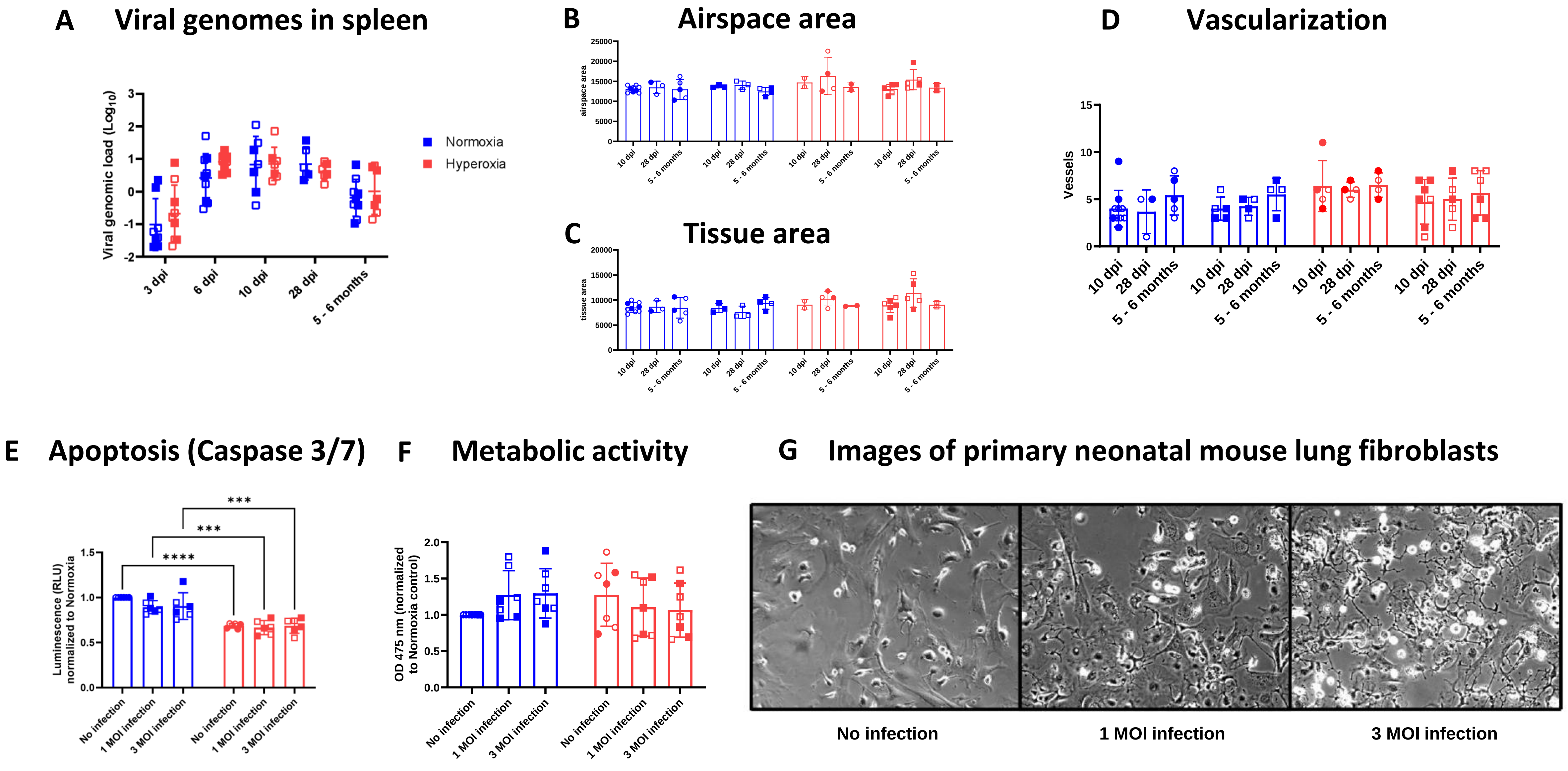
A Viral genomes in spleen
Viral genomic load (Log10)
3 dpi
6 dpi
10 dpi
28 dpi
5 - 6 months
Normoxia
Hyperoxia
B Airspace area
airspace area
C Tissue area
tissue area
10 dpi
28 dpi
5 - 6 months
D Vascularization
Vessels
E Apoptosis (Caspase 3/7)
Luminescence (RLU) normalized to Normoxia
****
***
***
No infection
1 MOI infection
3 MOI infection
F Metabolic activity
OD 475 nm (normalized to Normoxia control)
G Images of primary neonatal mouse lung fibroblasts
No infection
1 MOI infection
3 MOI infection

Supplementary Figure 1: Histopathologic and metabolic changes following MHV-68 infection with and without preceding hyperoxia in neonatal mice and primary neonatal mouse lung fibroblasts

A) Viral genomic load in the spleen. DNA was extracted from spleen pieces from female (n=3-4) and male mice (n=3-5). Viral DNA was quantified by qPCR. **B)** Airspace area measured in mice after hyperoxia exposure and MHV-68 infection (n=2-6 for female mice, and n=1-4 for male mice). H&E-stained lung histology slide images were converted into Bioquant software images. Three to four non-overlapping 100 μm fields were sampled from each section, intentionally avoiding the large airways and blood vessels. Using the software, airspace areas were measured. Two-way ANOVA test with multiple comparisons corrections. **C)** Tissue area measured in the lungs of adult mice after hyperoxia exposure and MHV-68 infection (n=2-6 for female mice, and n=1-3 for male mice). H&E-stained lung histology slide images were converted into Bioquant software images. Three to four nonoverlapping 100 μm fields were sampled from each section, intentionally avoiding the large airways and blood vessels. Using the software, tissue areas were measured. Two-way ANOVA test with multiple comparisons corrections. **D)** Vascularization in adult mice after MHV-68 infection and hyperoxia exposure in childhood. N=2-3 female and 2-3 male mice. H&E-stained lung histology slide images were converted into Bioquant software images. Three to four non-overlapping 100 μm fields were sampled from each section. Vessels were measured with the help of software. Two-way ANOVA test with multiple comparisons corrections. **E)** Caspase 3/7 activity after MHV-68 infection with or without prior exposure to hyperoxia. Mouse primary lung fibroblasts were isolated from n=3 female and n=3 male mice. Statistical analysis: Two-way ANOVA test with correction for multiple comparisons. *p-value < 0.05, **p-value < 0.01, ***p-value < 0.001, ****p-value < 0.0001. **F)** Metabolic activity after MHV-68 infection with or without prior exposure to hyperoxia. Mouse primary lung fibroblasts were isolated from n=4 female and n=3 male mice. Two-way ANOVA test with correction for multiple comparisons. **G)** Photomicrographs of primary neonatal mouse lung fibroblasts. Mouse primary fibroblasts were isolated from the lungs of newborn mice. Normoxia conditions without infection (left panel), 24 hours of MHV-68 infection with MOI of 1 (middle panel) and with MOI of 3 (right panel).

Data are always represented as mean $\pm$ SD. dpi: days post infection; open symbols: females; closed symbols: males.

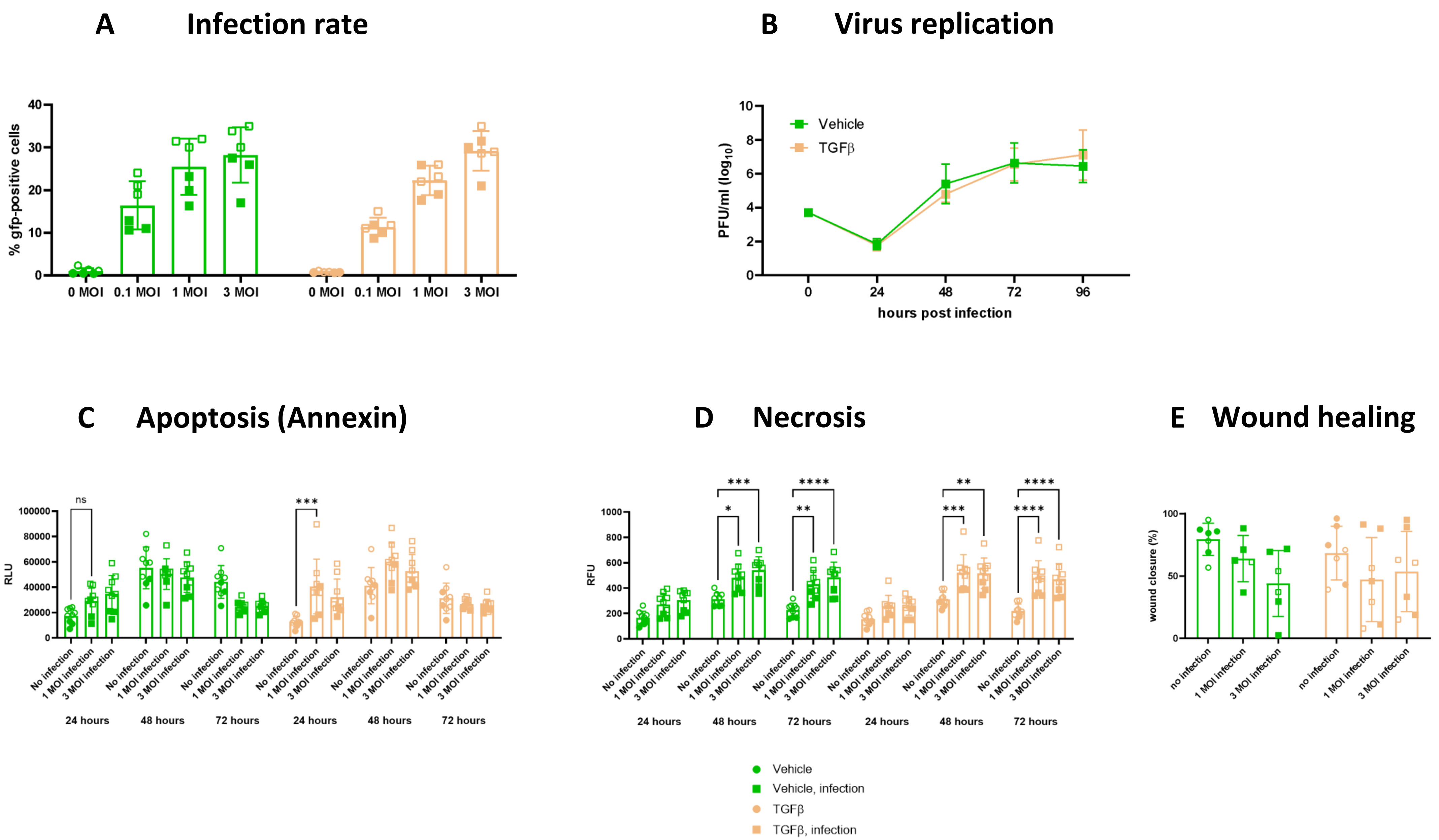
A Infection rate
% gfp-positive cells
0 MOI
0.1 MOI
1 MOI
3 MOI
B Virus replication
PFU/ml (log_{10})
Vehicle
TGFβ
hours post infection
C Apoptosis (Annexin)
RLU
ns
***
No infection
1 MOI infection
3 MOI infection
24 hours
48 hours
72 hours
D Necrosis
RFU
*
**
***
****
E Wound healing
wound closure (%)
no infection
1 MOI infection
3 MOI infection
Vehicle
Vehicle, infection
TGFβ
TGFβ, infection

Supplementary Figure 2: MHV-68 induced changes in primary neonatal mouse lung fibroblasts were not augmented by pre-treatment with TGFβ

A) MHV-68 infection rates in mouse primary lung fibroblasts with or without prior incubation with TGFβ for 24h. Primary neonatal mouse lung fibroblasts were isolated from the lungs of n=3 female and n=3 male newborn mice. Percentages of gfp-positive (= infected) cells are shown (mean ± SD). Vehicle treatment served as a control for TGFβ. Two-way ANOVA test with multiple comparisons correction was used for statistical analysis. **B)** MHV-68 replication in primary neonatal mouse lung fibroblasts after TGFβ treatment. Mouse primary fibroblasts were isolated from the lungs of n=5-6 female and n=5-6 male mice. Cells were infected with an MOI of 0.1, and virus titers were determined by Plaque assay at the indicated time points after infection. Vehicle treatment served as a control for TGFβ treatment. Two-way ANOVA test with multiple comparisons correction was used for statistical analysis. **C)** Apoptosis (Annexin V binding) after MHV-68 infection and TGFβ treatment. Mouse primary fibroblasts were isolated from the lungs of n=6 female and n=3 male mice. Vehicle treatment served as a control for TGFβ treatment. RLU: relative luminescence units. Two-way ANOVA test with multiple comparisons correction. *p-value < 0.05, **p-value < 0.01, ***p-value < 0.001, ****p-value < 0.0001. **D)** Necrosis after MHV-68 infection and TGFβ treatment. Primary neonatal mouse lung fibroblasts were isolated from the lungs of n=6 female and n=3 male mice. Vehicle treatment served as a control for TGFβ treatment. RFU: relative fluorescence units. Two-way ANOVA test with multiple comparisons correction. *p-value < 0.05, **p-value < 0.01, ***p-value < 0.001, ****p-value < 0.0001. **E)** Wound healing (“scratch” assay) after MHV-68 infection and TGFβ treatment. Primary neonatal mouse fibroblasts were isolated from the lungs of female (n=2-3) and male (n=3-4) mice. Vehicle treatment served as a control for TGFβ treatment accordingly. Statistical analysis: Two-way ANOVA test with correction for multiple comparisons. *p<0.05, **p<0.01, ***p<0.001, ****p<0.0001.

Data are always represented as mean ± SD. open symbols: females; closed symbols: males.

Body weights

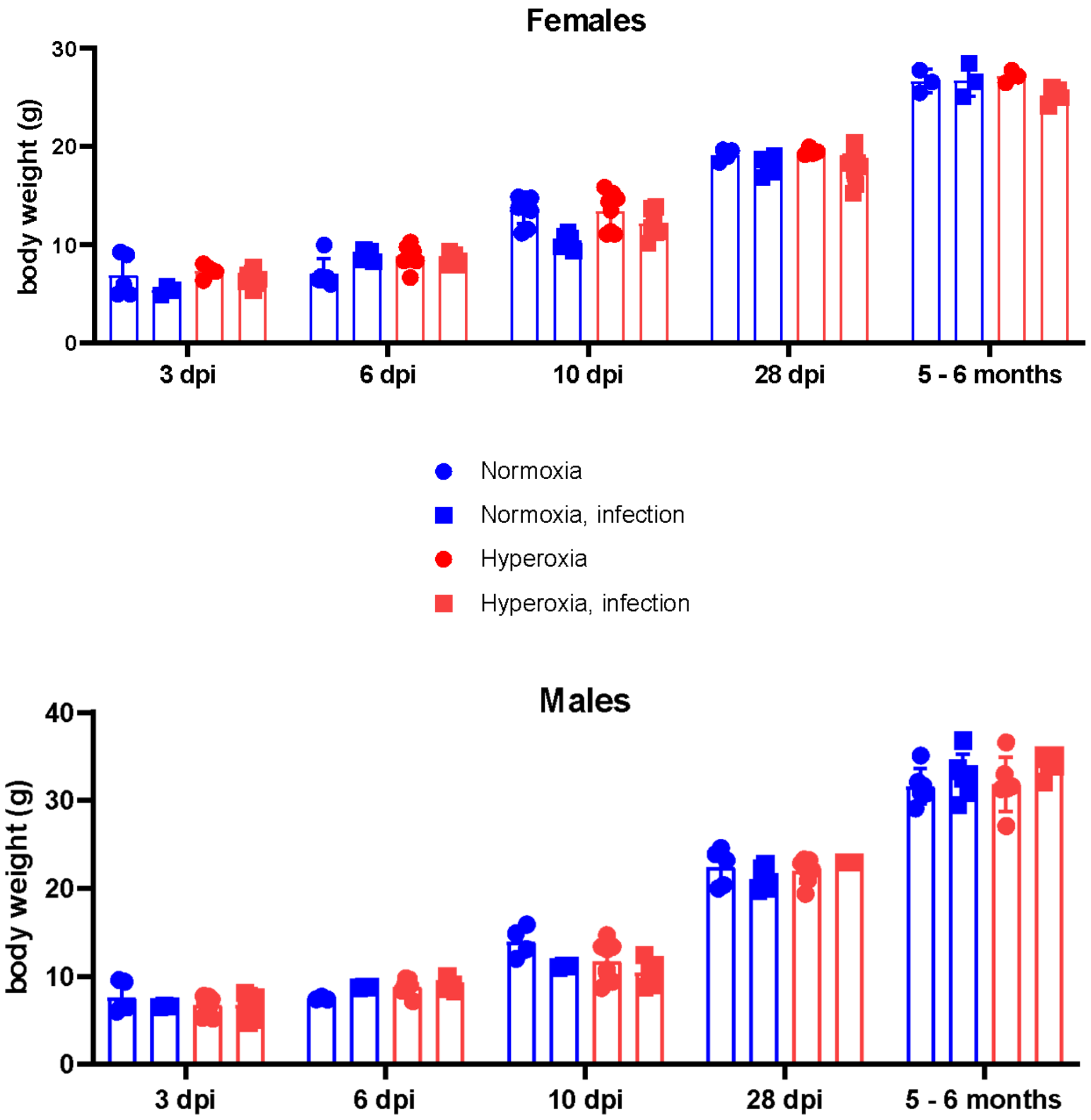

Supplementary Figure 3: Body weights of mice over the course of the study

Body weight measurements were taken on the day of sacrification from n=3-8 females (upper panel) and n=3-8 males (lower panel) over time for all four treatment groups.
