# Supplementary material for: Concerted action of gammaherpesvirus infection and moderate hyperoxia on lung structural development including effects on critical lung fibroblast functions in neonatal mice
Source: Mol Cell Pediatr. 2026 Aug 12;13:43. doi: 10.1186/s40348-026-00256-x (PMC13462024; doi:10.1186/s40348-026-00256-x)

Supplementary Figure: Original uncropped full-length Western blots to Figure 2I

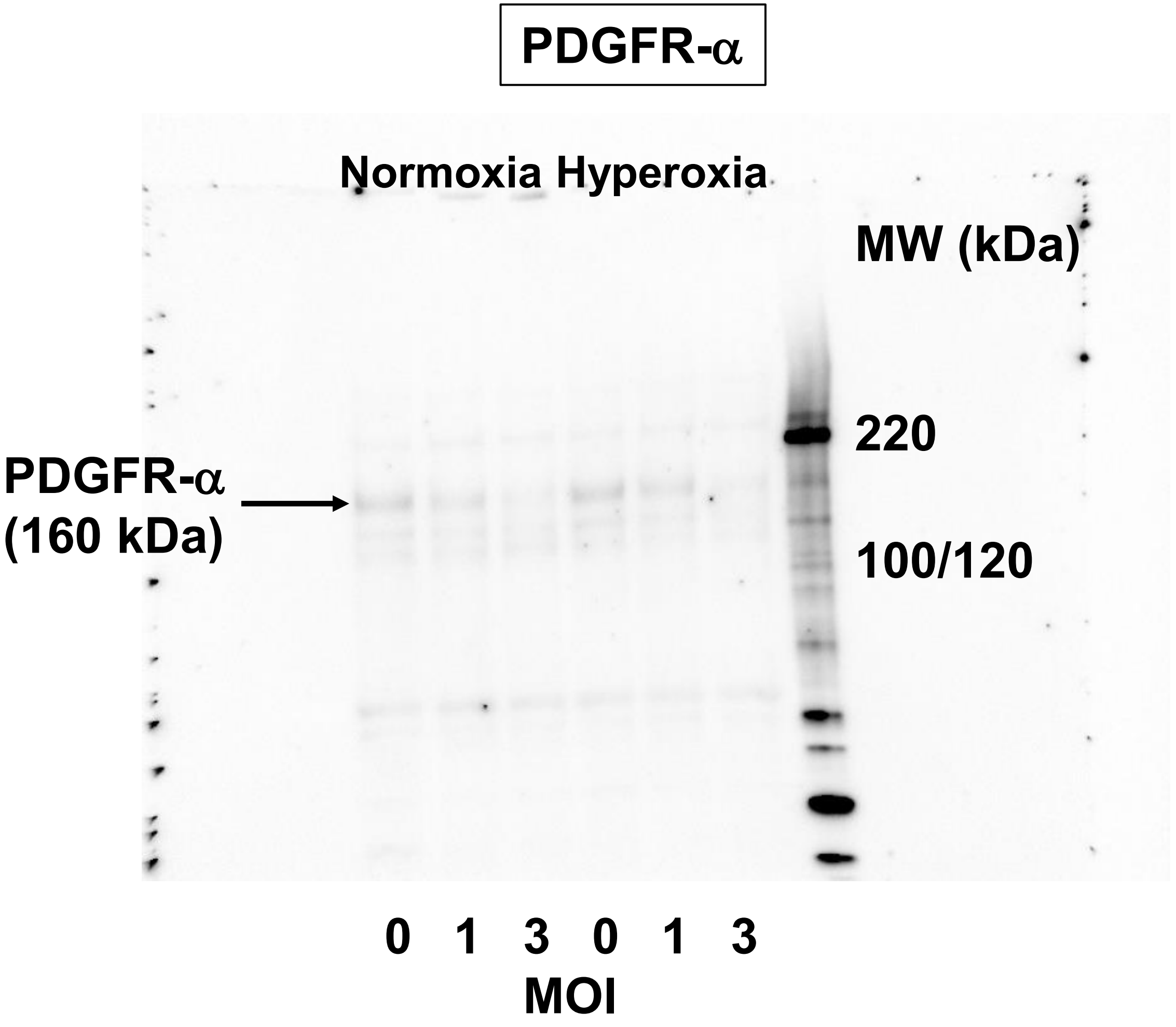

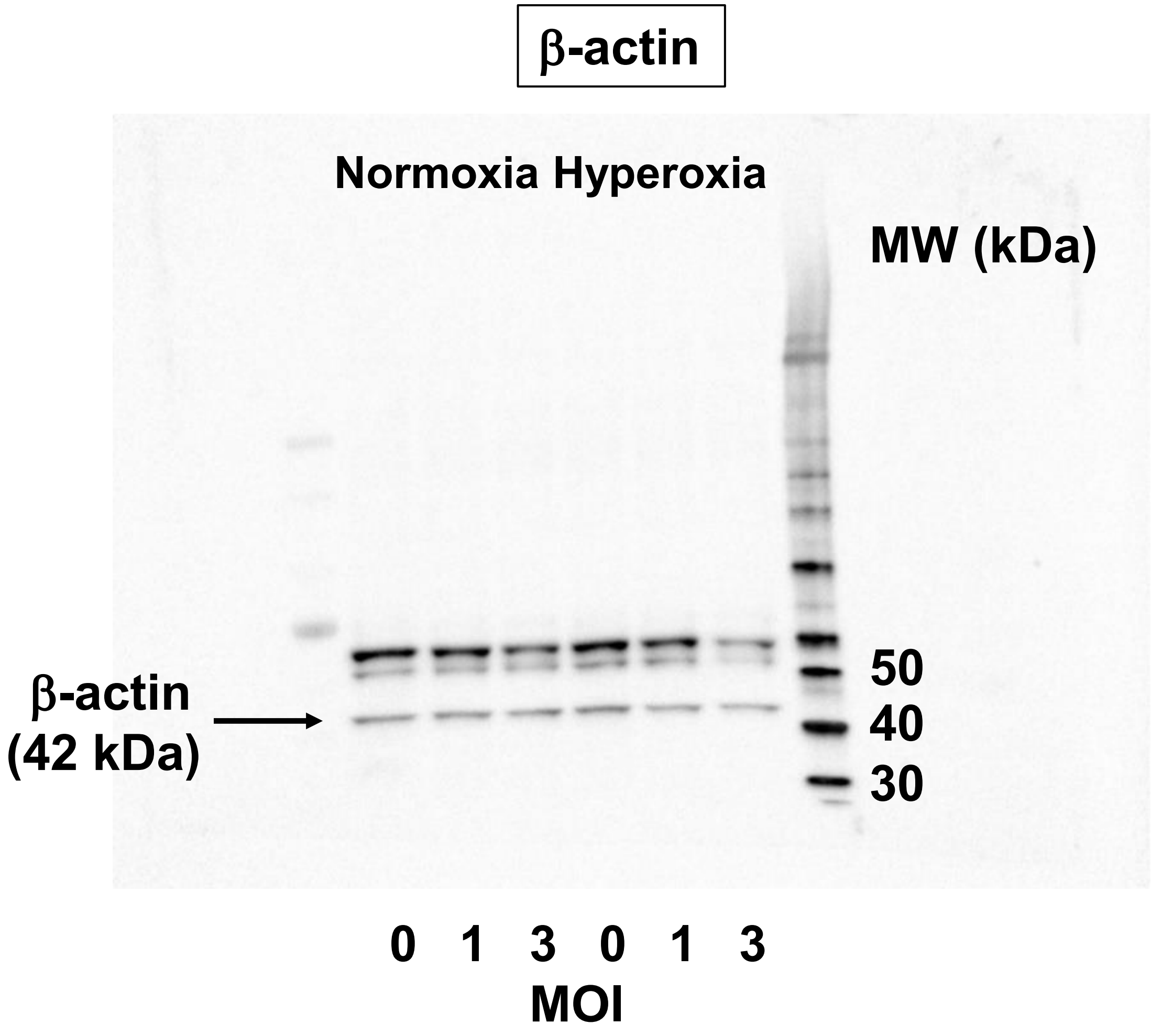

Supplement: Supplementary file 2 [file 40348_2026_256_MOESM2_ESM.pdf]
